# Monitoring of prostate-specific antigen in men with benign prostate enlargement receiving 5-alpha reductase inhibitors: a non-interventional, cross-sectional study of real-world practice of urologists in Spain and Brazil

**DOI:** 10.1186/s12894-025-01701-1

**Published:** 2025-01-31

**Authors:** Juan Manuel Palacios, Pratiksha Kapse, Vanessa Cortes, Marcio Augusto Averbeck, Alberto Budia Alba, Suryakant Somvanshi, Danilo Souza Lima da Costa Cruz, Fiona Pereira

**Affiliations:** 1 Global Medical Urology, GSK, Madrid, Spain; 2 Global Medical Urology, GSK, Mumbai, India; 3 Global Medical Infectious Diseases, GSK, Bogota, Colombia; 4 Department of Urology, Moinhos de Vento Hospital and Department of Urology, São Lucas Hospital – PUCRS, Porto Alegre, Brazil; 5 Department of Urology, La Fe University and Polytechnic Hospital, Valencia, Spain; 6 Dev Biostats, India Stats, GSK, Bangalore, India; 7 Department of Urology, State University of Rio de Janeiro, Rio de Janeiro, Brazil; 8 Brand and Integrated Research Solutions, IQVIA, London, UK

**Keywords:** 5-alpha reductase inhibitors, Benign prostatic hyperplasia, Benign prostate enlargement, Prostate-specific antigen, Real-world data

## Abstract

**Background:**

Inconsistent monitoring of prostate-specific antigen in patients receiving 5-alpha reductase inhibitors for lower urinary tract symptoms/benign prostate enlargement may affect prostate cancer outcomes. This study evaluated real-world practice among urologists treating patients receiving 5-alpha reductase inhibitors.

**Methods:**

This non-interventional, cross-sectional study collected data from urologists in Spain *(N* = 100) and Brazil *(N* = 100) via a self-reporting questionnaire and patient record forms. Endpoints included: frequency/methodology of prostate-specific antigen monitoring, concerns about the effect of 5-alpha reductase inhibitors on prostate-specific antigen monitoring, triggers of prostate biopsy, and concerns when switching 5-alpha reductase inhibitor formulation.

**Results:**

Over half of urologists monitored prostate-specific antigen every 6 months (Spain 59%, Brazil 58%). Preferred methods were the *“doubling rule”* (Spain 66%, Brazil 41%) and *“increase from nadir”* (Spain 28%, Brazil 43%). A minority of urologists monitored unadjusted values (Spain 3%, Brazil 11%) or did not monitor prostate-specific antigen (Spain 1%, Brazil 3%). Most urologists ranked the potential for 5-alpha reductase inhibitors to mask prostate cancer as their top concern (Spain 65%, Brazil 56%). The most selected trigger for prostate biopsy was *“if doubled (adjusted) prostate-specific antigen level after 6 months of treatment is* > *4 ng/mL”* (Spain 39%, Brazil 37%). Many urologists were moderately/very concerned about the effect on prostate-specific antigen when switching 5-alpha reductase inhibitor formulation.

**Conclusions:**

An unmet need exists for standard guidance and continuous education to support optimal monitoring and interpretation of prostate-specific antigen in patients with lower urinary tract symptoms/benign prostate enlargement treated with 5-alpha reductase inhibitors.

**Supplementary Information:**

The online version contains supplementary material available at 10.1186/s12894-025-01701-1.

## Background

Five-alpha reductase inhibitors (5ARIs) alone or in combination with alpha-adrenergic antagonists (α-blockers) are indicated to treat lower urinary tract symptoms due to benign prostate enlargement (LUTS/BPE) and reduce the risk of acute urinary retention or prostatic-related surgery [1–4]. Clinical guidelines have incorporated evidence from long-term clinical studies [5–7] to recommend 5ARIs as a treatment for patients with LUTS/BPE [8–11].

LUTS/BPE and prostate cancer may coexist, and levels of prostate-specific antigen (PSA) in blood can be used to inform the risk of LUTS/BPE disease progression and screen for prostate cancer [12]. PSA testing for early detection of prostate cancer is supported by clinical guidelines [8–11]. 5ARIs lower PSA levels by reducing the size of the glandular epithelial component of the prostate, which is responsible for PSA production [8]. This effect on PSA levels has created concern that 5ARI treatment may mask the early detection of prostate cancer. It has been speculated that improper PSA monitoring and interpretation in patients receiving 5ARIs could lead to delays in prostate cancer diagnosis, which may result in advanced disease and worse clinical outcomes [13]. PSA levels can also be impacted by switching to alternative 5ARIs with different formulas (e.g. switching between finasteride and dutasteride, or between branded and generic 5ARI formulations) [14].

Given the clinical relevance of appropriately monitoring and interpreting PSA in patients with LUTS/BPE receiving 5ARIs, there is a need to understand current practices. The overall aim of this study was to evaluate real-world practice among urologists monitoring PSA levels in patients with LUTS/BPE receiving 5ARIs. In doing so, it provides crucial information to identify and address gaps between evidence-based guidelines and real-world clinical practice.

## Methods

### Study design, objectives and data collection

This non-interventional, cross-sectional study collected data from urologists in Spain and Brazil. These countries were chosen based on the prevalence of benign prostatic hyperplasia (BPH) and annual 5ARI usage. In Spain, the prevalence of BPH was estimated to be 11.8% in men > 40 years and approximately 30% in men older than 70 [15]. In Brazil, the prevalence of BPH in men aged 60‒90 years was estimated to be 53.5% [16]. Spain and Brazil have relatively high usage of 5ARI globally [17–19]. Data were collected through an online, self-reporting survey comprising a questionnaire and patient record forms (PRFs; Supplementary Methods) for specific patients in the urologist’s care, all completed by the urologist. All questions concerned patients with LUTS/BPE treated with 5ARI monotherapy or in combination with α-blockers.

The objectives of this study were to: (I) investigate the frequency and methodology of PSA monitoring in patients with LUTS/BPE receiving 5ARIs; (II) gain insights into the concerns of urologists about the effect of 5ARI treatment on PSA monitoring related to prostate cancer screening; (III) investigate how changes in PSA are interpreted when considering a biopsy for prostate cancer in patients with LUTS/BPE receiving 5ARIs; and (IV) gain insights into possible urologist concerns when switching between 5ARI formulations.

The questionnaire comprised multiple-choice, open-ended, ranking and rating parameters developed empirically through review of LUTS/BPE literature and health questionnaires. Urologists were asked to complete a PRF for each of their two most recent patients with LUTS/BPE (aged ≥ 50 years and receiving 5ARI treatment initiated by the urologist in the year prior to completing the questionnaire) to better assess the current real-world practice of managing patients with LUTS/BPE. To aid accuracy, urologists were asked to consult their charts first, then answer all questions in the PRF one patient at a time.

Questionnaire and PRF data relevant to PSA monitoring are reported here; relevant questions are outlined in Supplementary Table 1. For each variable collected, a pre-defined list of categories was provided for participants to choose from. The study documents were reviewed and approved by institutional review boards, in accordance with the ethical regulations of each country.

### Sample characteristics

Urologists were recruited from Spain and Brazil *(N* = 100 from each country; February–April 2023). Participants were selected at random from country-specific urologist panels, including the IQVIA (formerly the Intercontinental Medical statistics (IMS) Health and Quintiles) network. IQVIA is a provider of advanced analytics, technology solutions, and clinical research services. Participants gave their consent to be contacted for research studies.

Eligible urologists fulfilled the following inclusion criteria: 5‒35 years of clinical experience, ≥ 60% of their time in direct patient care, and having personally seen and managed ≥ 10 patients with LUTS/BPE in the month prior to completing the questionnaire. Respondents were asked 2–3 linked questions as quality control to address the possibility of misclassification; responses were checked to ensure the questions were answered in good faith, free-text answers were appropriate, and answers did not contradict themselves across the questionnaire. Respondents were excluded if they completed the questionnaire in less than half of the average time taken across all respondents.

Considering the nature of this study, sample size was based on a feasibility assessment with advice from an expert on BPH with extensive experience on the prevalence of LUTS/BPE and the patient population. The Power Analysis Software was used to determine if the sample size was adequate, and it determined a margin of error of 7%. Therefore, sample size was considered adequate to support quantitative analysis in this study.

At least 350 urologists in each country were contacted to achieve the required sample size. The study recruited ~ 5% additional respondents to ensure a total sample size of 200 urologists and 400 PRFs were available for data analysis after exclusions.

### Questionnaire and PRFs

Urologists were provided with a link to an online screening tool and, if eligibility criteria were met, were taken through to the online questionnaire. Both the urologists and the patients described in the PRFs remained anonymous.

### Statistical analysis

The analysis was primarily descriptive in nature and included a correlation analysis approach to address one of the secondary objectives. Data were analyzed separately for each country to allow for country-specific treatment patterns to be examined. Furthermore, Brazil and Spain utilize different levels of government and private healthcare sector involvement. Drawing conclusions from comparing these two countries would result in inaccurate deductions. Thus, each country was analyzed independently. Frequencies and percentages of responses for a given category were presented for categorical and ordinal variables. Descriptive statistics were provided for frequency and methodology of PSA testing, concerns around effect of 5ARIs on PSA monitoring/interpretation, biopsy triggers, and treatment switching concerns. All analyses were performed using IBM SPSS statistics, version 23.

## Results

### Respondent characteristics

Overall, 100 urologists from each country were enrolled and completed the questionnaire. Supplementary Table 2 provides an overview of the characteristics of urologists who participated in the study. On average (standard deviation [SD]), urologists had > 10 years’ experience (Spain 16 years [7.8], Brazil 14 years [8.8]). In Spain, most urologists (87%) worked in the public setting, while in Brazil, most worked in the private setting (65%). Urologists in Spain saw an average of 149 men with LUTS/BPE per month, of whom an average of 80 (54%) were treated with 5ARIs (monotherapy or in combination with an α-blocker). In Brazil, the average monthly LUTS/BPE caseload was 68 patients, of whom an average of 54 (79%) were treated with 5ARIs.

A total of 400 PRFs were collected (200 from each country). In both countries, approximately half of patients were aged 55–64 years, with numerically more patients in younger age groups in Brazil versus Spain (Table 1). In both countries, approximately half of patients were diagnosed with LUTS/BPE > 12 months ago (Spain 44%, Brazil 52%). Most patients had comorbidities, such as hypertension (Spain 67%, Brazil 81%) and diabetes (Spain 46%, Brazil 49%). Sexual dysfunction was present in 27% of patients in Spain and 35% in Brazil. Based on either clinical assessment or international prostate symptom scores (IPSS), most patients captured via the PRFs had *“moderate”* disease severity.

**Table 1:** Demographics and disease characteristics of the patients included in the PRFs

Patient characteristic, n (%)	Spain (*N* = 200)	Brazil (*N* = 200)
Age, years		
50–54	9 (5)	20 (10)
55–59	41 (21)	42 (21)
60–64	51 (26)	51 (26)
65–69	28 (14)	35 (18)
70–74	41 (21)	30 (15)
75–79	19 (10)	12 (6)
≥ 80	11 (6)	10 (5)
Time since diagnosis		
< 7 months	55 (28)	52 (26)
7–12 months	58 (29)	45 (23)
> 12 months	87 (44)	103 (52)
Comorbidities		
Hypertension	133 (67)	162 (81)
Diabetes	92 (46)	98 (49)
Dyslipidemia	91 (46)	65 (33)
Overweight/obesity	73 (37)	78 (39)
Cardiovascular disease	55 (28)	40 (20)
Sexual dysfunction	53 (27)	69 (35)
Non-prostatic malignancy	4 (2)	1 (0.5)
Neurological condition	4 (2)	0 (0)
No comorbidities	18 (9)	17 (9)
Type of sexual dysfunction	*N* = 53	*N* = 69
Erectile dysfunction	46 (87)	63 (91)
Ejaculatory dysfunction	5 (9)	8 (12)
Negative impact on libido/sexual desire	4 (8)	16 (23)
Negative impact on global sexual function	5 (9)	10 (15)
Risk categorization when 5ARI was initiated	*N* = 200	*N* = 200
At risk of progression	163 (82)	176 (88)
Not at risk of progression	37 (19)	24 (12)
Symptom severity based on IPSS	*N* = 116	*N* = 51
Severe	45 (39)	13 (26)
Moderate	68 (59)	35 (69)
Mild	3 (3)	3 (6)
Symptom severity per clinical assessment	*N* = 81	*N* = 145
Severe	30 (37)	41 (28)
Moderate	49 (61)	94 (65)
Mild	2 (3)	10 (7)

Percentages may not total 100% for each characteristic due to rounding. Each country was analyzed independently in this study

*5ARI* 5-alpha reductase inhibitor, *IPSS* international prostate symptom score, *PRF* patient record form

### Frequency and methodology of PSA monitoring

Over half of urologists reported monitoring PSA levels every 6 months (Spain 59%, Brazil 58%). One-third of urologists reported monitoring PSA levels every 12 months or less (Spain 30%, Brazil 34%). A small number of urologists (5% in both countries) reported monitoring PSA levels every 3 months, or not monitoring PSA regularly but on a need basis only (Spain 6%, Brazil 3%).

**Fig. 1:**
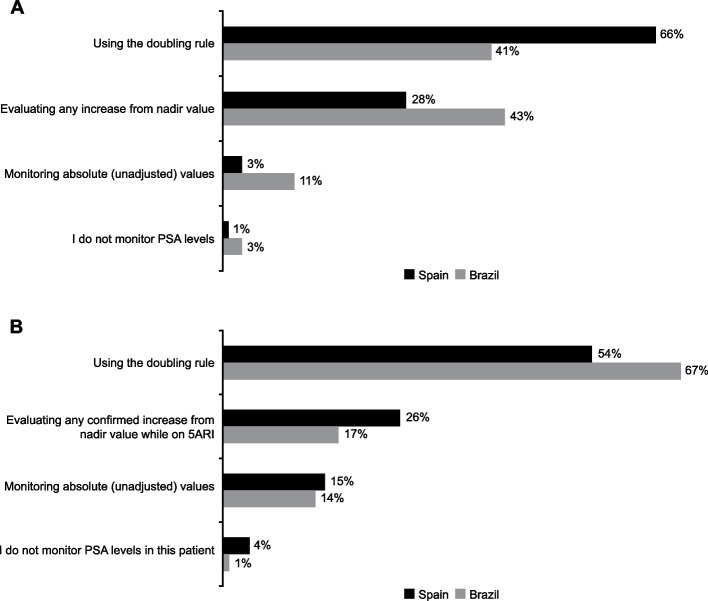
Method of PSA monitoring in patients with LUTS/BPE treated with 5ARIs. Results according to (**A**) the questionnaire and (**B**) PRFs. 5ARI, 5-alpha reductase inhibitor; LUTS/BPE, lower urinary tract symptoms/benign prostate enlargement; PRF, patient record form; PSA, prostate-specific antigen. Each country was analyzed independently in this study

When asked about their method of interpreting PSA levels, 66% of urologists in Spain chose *“using the doubling rule”*, 28% chose *“evaluating any increase from nadir value”*, 3% chose *“monitoring absolute (unadjusted) values”*, and 1% chose *“I do not monitor PSA levels”.* In the PRFs, the respective percentages for these options were 54%, 26%, 15%, and 4%. In Brazil, 41% selected *“using the doubling rule”*, 43% of urologists selected *“evaluating any increase from nadir value”*, 11% selected *“monitoring absolute (unadjusted) values”*, and 3% selected *“I do not monitor PSA levels”.* In the PRFs, the respective percentages were 67%, 17%, 14%, and 1% (Fig. 1).

### Urologist concerns about the effect of 5ARI treatment on PSA monitoring

Participants were asked to indicate which of the following concerns that urologists might have when monitoring PSA in patients receiving 5ARIs were most important. The most frequently top-ranked response was *“concerned about the effect of 5ARIs on PSA levels that may mask prostate cancer”* (Spain 65%, Brazil 56%). The next most frequently top-ranked response was *“concerned about how to interpret and monitor PSA in men receiving 5ARIs for prostate cancer screening”* (Spain 18%, Brazil 39%). *“Concerned about impact on PSA when changing between 5ARI brands and branded versus generic”* was ranked as the highest concern by 14% of urologists in Spain and 3% in Brazil.

### Triggers for prostate biopsy

**Fig. 2:**
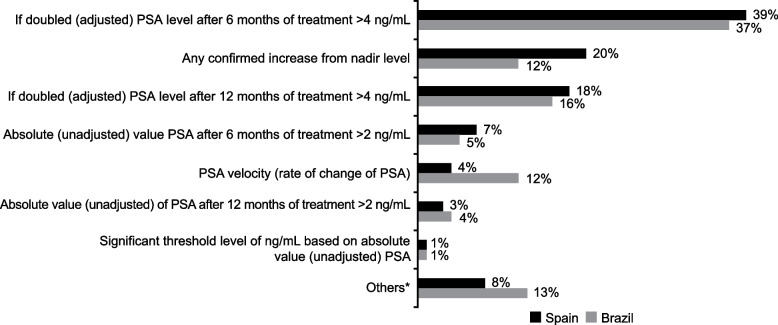
Triggers for prostate biopsy in patients with LUTS/BPE treated with 5ARIs. *A free-text box was provided so participants could give other triggers for prostate biopsy not included in the question. 5ARI, 5-alpha reductase inhibitor; LUTS/BPE, lower urinary tract symptoms/benign prostate enlargement; PSA, prostate-specific antigen. Each country was analyzed independently in this study

The most selected trigger for prostate biopsy in both countries was *“if doubled (adjusted) PSA level after 6 months of treatment is* > *4 ng/mL”* (Spain 39%, Brazil 37%; Fig. 2). The next most common response from urologists in Spain was *“any confirmed increase from nadir level”* (20%) and in Brazil was *“if doubled (adjusted) PSA level after 12 months of treatment is* > *4 ng/mL”* (16%). Among those who selected *“PSA velocity”* (the rate of change of PSA), the range of thresholds specified by the urologists was typically between 0.7–1 ng/mL/year.

### Urologist concerns when switching 5ARI formulation

**Fig. 3:**
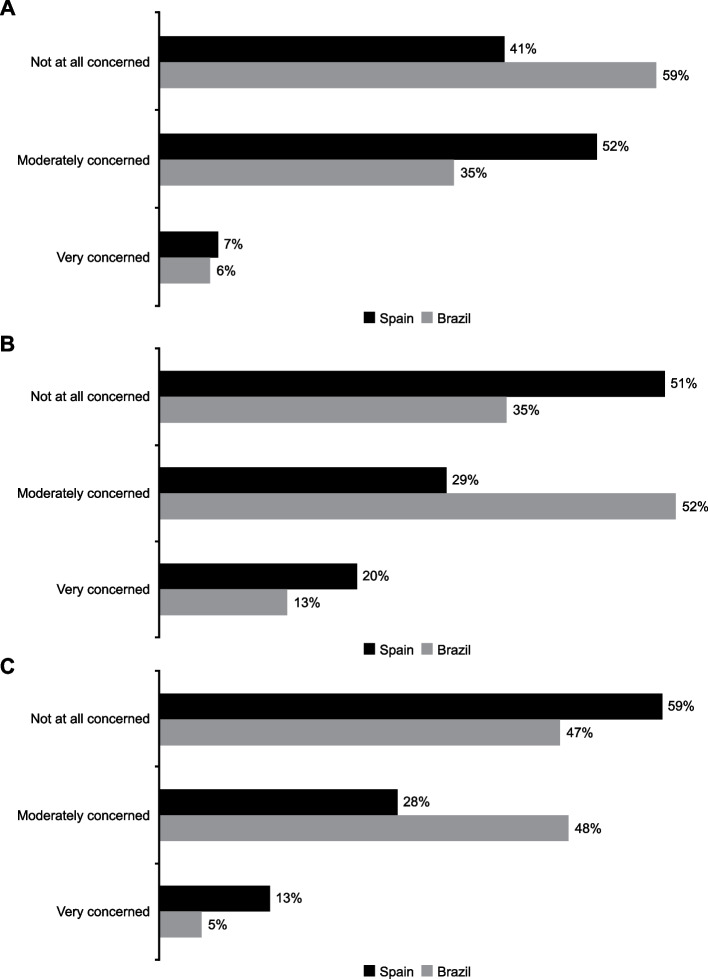
Key concerns that urologists have regarding the impact on PSA levels when changing medications. Changing from (**A**) dutasteride to finasteride or vice versa (**B**) branded to generic medications, and (**C**) changing between generic options of 5ARIs. 5ARI, 5-alpha reductase inhibitor; PSA, prostate-specific antigen. Each country was analyzed independently in this study

Over half of urologists in Spain (52%) reported they were *“moderately concerned”* about the impact on PSA levels when changing between 5ARI molecules (dutasteride to finasteride or vice versa). Conversely, most urologists in Brazil (59%) selected the option *“not at all concerned”*. Fewer than one in 10 urologists in both countries were *“very concerned”* about the impact of switching 5ARI molecules on PSA levels (Spain 7%, Brazil 6%; Fig. 3A).

On changing from branded to generic 5ARIs, 51%, 29%, and 20% of urologists in Spain reported being *“not at all concerned”*, *“moderately concerned”*, and *“very concerned”*, respectively, about the impact on PSA levels (Fig. 3B). Responses from urologists in Brazil showed 35%, 52%, and 13% reporting *“not at all concerned”*, *“moderately concerned”*, and *“very concerned”*, respectively.

Regarding the impact on PSA management of changing between generic medications, 59% of urologists in Spain and 47% in Brazil reported they were *“not at all concerned”*, 28% in Spain and 48% in Brazil answered *“moderately concerned”*, and 13% in Spain and 5% in Brazil selected *“very concerned”* (Fig. 3C).

## Discussion

Treatment with 5ARIs in patients with LUTS/BPE lowers PSA levels by approximately 50% after 6 months of treatment, even when prostate cancer is present [20, 21]. This has led to the concern that 5ARI treatment may mask early prostate cancer detection [13]. This is reflected in our study, where over half of the urologists surveyed reported concerns about the potential masking effect of 5ARIs on prostate cancer. Most urologists considered regular PSA monitoring important in men with LUTS/BPE treated with 5ARIs, primarily for the purpose of identifying prostate cancer, and around 95% of urologists reported monitoring PSA at regular intervals.

The effect of 5ARIs on PSA levels has led to changes in monitoring and interpreting PSA for prostate cancer detection. Currently, there are two main methods to do so in men receiving 5ARIs: the *“doubling rule”* and *“increase from nadir”*. The *“doubling rule”* is a method of adjusting PSA levels to account for the impact of 5ARIs; after 6 months of treatment with 5ARIs, PSA values are doubled and then compared with normal ranges from untreated patients [22]. The second method involves establishing each patient’s lowest PSA level (nadir) over the course of 6 months of treatment with 5ARIs, then monitoring for any confirmed increase from that baseline as a potential indicator of prostate cancer [3, 23]. In the REDUCE study, the *“increase from nadir”* method was shown to maintain the sensitivity of PSA levels as a cancer biomarker in men receiving dutasteride [3]. In a prospective, multicenter study of 203 men with BPH receiving dutasteride in Japan, a > 10% increase in PSA levels after 1 year of treatment significantly predicted the risk of a prostate cancer diagnosis [24].

This study showed that both *“increase from nadir”* and the *“doubling rule”* are being used widely in real-world clinical practice; the *“doubling rule”* was more common, despite being considered less reliable [25]. Notably, according to the PRFs, 15% of urologists in Spain and 14% in Brazil monitor absolute, unadjusted PSA values and 4% of urologists in Spain and 1% in Brazil do not monitor PSA levels at all, which is concerning. It is unsurprising that uptake of *“increase from nadir”* method has been limited given the inconsistencies in 5ARI labeling information and guidelines. While the European Union (EU) Summary of Product Characteristics (SmPC) for dutasteride has been updated to recommend using the *“increase from nadir”* method [4], the SmPC for finasteride still recommends the *“doubling rule”* [2, 26], whilst the Food and Drug Administration (FDA) labels for dutasteride [27] and finasteride [26] include instructions for both PSA monitoring methods. Furthermore, there is a lack of consensus and clarity around the most appropriate method to use in the published guidelines [10, 11]. There is an ongoing need to inform urologists of current best practices and the favorable sensitivity of the *“increase from nadir”* method when evaluating PSA levels in patients treated with 5ARIs.

The PSA threshold that most urologists used to trigger a prostate biopsy was consistent between countries. Around four in 10 urologists (Spain 39%, Brazil 37%) reported the trigger to be *“if doubled (adjusted) PSA level after 6 months of treatment is* > *4 ng/mL”*. However, the second most chosen option differed between countries: 20% of urologists in Spain chose *“any confirmed increase from nadir level”*, while in Brazil, 16% selected *“if doubled (adjusted) PSA level after 12 months of treatment is* > *4 ng/mL”*. In the general population, a PSA level of 4 ng/mL is commonly used by urologists and in primary care settings, since this threshold was used in the Prostate Cancer Prevention Trial and REDUCE trial [20, 28]; however, the optimal threshold for biopsy in clinical practice is debatable and should take other clinical factors into consideration.

Switching between 5ARIs can substantially affect PSA levels, potentially impacting whether a biopsy is undertaken [14]; this puts additional burden on urologists as they optimize treatment while considering fluctuating PSA results and their impact on prostate cancer diagnosis. This study highlights that a higher proportion of urologists in Brazil were moderately concerned about switching from branded to generic medications (52%) or from generic to branded medications (48%) versus Spain (29% and 28%, respectively). However, a higher proportion of urologists in Spain were moderately concerned when changing patients from dutasteride to finasteride or vice versa (52%) compared with Brazil (35%). More evidence is needed to understand the effects of switching 5ARIs on PSA levels and to determine how urologists should account for this to interpret any shifts that may indicate that a biopsy is required.

The impact of treatment with 5ARIs on prostate cancer diagnosis is complex. Although some database analyses have suggested that the use of 5ARIs can lead to delayed diagnosis and worse cancer-specific mortality [13], it is likely that these negative outcomes resulted from use of unadjusted PSA levels, potentially leading to incorrect PSA interpretation. More recent studies show no association between treatment with 5ARIs and increased prostate cancer mortality in men without a previous prostate cancer diagnosis [22].

A strength of this study is the use of real-world data representative of real-world clinical practice, suggesting that the learnings from this study can be generalized to a wider population. A limitation of this study is that data were self-reported by urologists and may be prone to recall bias; however, the combined questionnaire and PRF approach may have reduced the self-reporting bias. Another limitation was the potential for selection bias, as urologist inclusion was dependent on internet availability and meeting the inclusion criteria. Additionally, data were only collected from two countries with high rates of LUTS/BPE; therefore, the results may not be representative from an international perspective. These results do, however, help identify the real-world practice of urologists in two countries where 5ARI use is high, which could help inform 5ARI prescribing, and PSA monitoring practices in countries where 5ARI use is less common. Future studies should aim to understand how clinicians use PSA density to interpret PSA levels and inform prescribing.

## Conclusions

This analysis revealed that a large proportion of urologists in Spain and Brazil have not adopted the best practice *“increase from nadir”* method for PSA monitoring and interpretation, and some urologists monitor unadjusted PSA values or do not monitor PSA levels at all; thus, educational efforts should continue to emphasize the appropriate method of PSA monitoring and interpretation. Urologists were concerned about the effect of 5ARIs on masking prostate cancer when monitoring PSA levels, highlighting a need to provide additional data and education on the latest evidence, which would allow urologists to make informed choices when treating and monitoring patients with LUTS/BPE receiving 5ARIs [24, 29–34]. Future studies should consider the role of PSA density in patients with LUTS/BPE receiving 5ARIs.

## Supplementary Information

Supplementary Material 1: Supplementary Methods. Supplementary Table 1. Questions used in questionnaire and PRFs to address the study objectives. Supplementary Table 2. Characteristics of urologists who completed the questionnaire.

## Data Availability

All data generated or analyzed during this study are included in this article. Further enquiries can be directed to the corresponding author.

## Abbreviations

5ARI: 5-alpha reductase inhibitor
BPH: Benign prostatic hyperplasia
EU: European Union
FDA: Food and Drug Administration
IPSS: International prostate symptom score
LUTS/BPE: Lower urinary tract symptoms/benign prostate enlargement
PRF: Patient record form
PSA: Prostate-specific antigen
SD: Standard deviation
