# Supplementary material for: Monitoring of prostate-specific antigen in men with benign prostate enlargement receiving 5-alpha reductase inhibitors: a non-interventional, cross-sectional study of real-world practice of urologists in Spain and Brazil
Source: BMC Urol. 2025 Jan 31;25:22. doi: 10.1186/s12894-025-01701-1 (PMC11786547; doi:10.1186/s12894-025-01701-1)
Supplement: Supplementary file 1 — Supplementary Material 1: Supplementary Methods. Supplementary Table 1. Questions used in questionnaire and PRFs to address the study objectives. Supplementary Table 2. Characteristics of urologists who completed the questionnaire. [file 12894_2025_1701_MOESM1_ESM.docx]

**SUPPORTING INFORMATION**

**Real-world practice of urologists in Spain and Brazil: Monitoring of prostate-specific antigen in men with benign prostatic enlargement receiving 5-alpha reductase inhibitors**

SUPPLEMENTARY METHODS

Questionnaire: Selected questions from urologist questionnaire. Note: Questions have been renumbered

**Q1.**

**What concerns you the most when monitoring PSA in BPH patients receiving 5ARIs (in monotherapy or in combination with alpha blockers)?**

**Please rank in order of importance, with 1 being most important, 2 being second most important, and so on.**

| **Reasons for PSA concerns** |
| --- |
| Concerned about the effect of 5ARIs on PSA levels that may mask prostate cancer |
| Concerned about how to interpret and monitor PSA in patients receiving 5ARIs for PCa screening |
| Concerned about impact on PSA when changing between 5ARI brands and branded versus generic |
| Other (please specify) _______ |
| Not concerned about PSA monitoring in BPH patients on 5ARIs |

5ARI, 5-alpha reductase inhibitor; BPH, benign prostatic hyperplasia; PCa, prostate cancer; PSA, prostate-specific antigen.

**Q2.**

**How often do you monitor PSA levels in your BPH patients currently being treated with 5ARIs (in monotherapy or in combination with alpha blockers)?**

| **Frequency of PSA testing** |
| --- |
| Every 3 months (once a quarter) |
| Every 6 months |
| Every 12 months (once a year) or less frequently |
| Not regularly but as on need basis only |
| Do not monitor PSA levels |

5ARI, 5-alpha reductase inhibitor; BPH, benign prostatic hyperplasia; PSA, prostate-specific antigen.

**Q3.**

**How do you monitor PSA levels in your BPH patients currently being treated with 5ARIs (in monotherapy or in combination with alpha blockers)?**

| **PSA interpretation** |
| --- |
| Using the doubling rule |
| Evaluating any increase from nadir value |
| Monitoring absolute (unadjusted) values |
| I do not monitor PSA levels |
| Other (please specify) _______ |

5ARI, 5-alpha reductase inhibitor; BPH, benign prostatic hyperplasia; PSA, prostate-specific antigen.

**Q4.**

**Which of the following triggers do you consider for prostate biopsy for prostate cancer suspicion in your BPH patients currently being treated with 5ARIs (in monotherapy or in combination with alpha blockers)?**

| **Triggers for biopsy** |
| --- |
| Any increase from nadir level |
| If doubled (adjusted) PSA level after 6 months of treatment >4 ng/mL |
| If doubled (adjusted) PSA level after 12 months of treatment >4 ng/mL |
| Absolute (unadjusted) value of PSA after 6 months of treatment >2 ng/mL |
| Absolute (unadjusted) value of PSA after 12 months of treatment >2 ng/mL |
| Significant threshold level of _________ng/mL (open text box) based on absolute (unadjusted) value of PSA |
| PSA velocity (rate of change of PSA) |
| Please indicate significant threshold level for PSA velocity:________ (open text box) |
| Other (please specify) _______ |

5ARI, 5-alpha reductase inhibitor; BPH, benign prostatic hyperplasia; PSA, prostate-specific antigen.

**Q5.**

**What is your level of concern about the following aspects of PSA management in BPH patients currently being treated with 5ARIs (in monotherapy or in combination with alpha blockers)?**

1. **Changing between 5ARI molecules—dutasteride to finasteride or vice versa**
2. **Changing from branded to generic options of 5ARIs (in monotherapy or in combination with alpha blockers)**

| **PSA concerns in 5ARIs** | **Very concerned** | **Moderately concerned** | **Not at all concerned** |
| --- | --- | --- | --- |
| Changing between 5ARI molecules—dutasteride to finasteride or vice versa |  |  |  |
| Changing from branded to generic options of 5ARIs |  |  |  |
| Changing between generic options of 5ARIs |  |  |  |

5ARI, 5-alpha reductase inhibitor; BPH, benign prostatic hyperplasia; PSA, prostate-specific antigen.

Patient record form (PRF): Selected questions from urologist patient record forms. Note: Questions have been renumbered; the full set of PRF questions is available in the study protocol.

**P1. What is the age of this patient?**

| **Age** |
| --- |
| 50–54 years old |
| 55–59 years old |
| 60–64 years old |
| 65–69 years old |
| 70–74 years old |
| 75–79 years old |
| More than 80 years old |

**P2. When was this patient first diagnosed with BPH?**

| **Time since diagnosis** |
| --- |
| In the last 6 months |
| 7–12 months ago |
| More than 12 months ago |

BPH, benign prostatic hyperplasia.

**P3. Which comorbidities are present in this patient?**

| **Comorbidities** |
| --- |
| Hypertension |
| Diabetes |
| Cardiovascular disease |
| Sexual dysfunction |
| Erectile dysfunction  Ejaculatory dysfunction  Negative impact on libido/sexual desire  Negative impact on global sexual function  Other (please specify) __________ |
| Neurological condition |
| Type of neurological condition ____________ |
| Overweight/obesity |
| Dyslipidemia |
| Non-prostatic malignancy |
| No comorbidities |
| Other (please specify) _______ |

**P4. How did you interpret PSA values in this patient?**

| **PSA interpretation** |
| --- |
| Using the doubling rule |
| Evaluating any confirmed increase from nadir value while on 5ARI |
| Monitoring absolute (unadjusted) values |
| I did not monitor PSA levels in this patient |
| Other (please specify) _______ |

**P4a. You mentioned that you use the doubling rule to interpret PSA level in this patient. Can you please specify which option below best describes your approach with this patient?**

| **PSA interpretation** |
| --- |
| Correction of PSA values (doubling of the PSA value) after 6 months of therapy |
| Correction of PSA values (doubling of the PSA value) after 1 year of therapy |
| Other (please specify) _______ |

5ARI, 5-alpha reductase inhibitor; PSA, prostate-specific antigen.

**SUPPLEMENTARY TABLE 1** Questions used in questionnaire and PRFs to address the study objectives.

| **Study objective** | **Questionnaire** | **PRF** |
| --- | --- | --- |
| How frequently do urologists monitor PSA in patients with LUTS/BPE taking 5ARIs for progression and what methods of PSA monitoring do they use? | Q2  Q3 | P4 P4a |
| What concerns do urologists have regarding the effect that 5ARIs have on PSA levels that may mask prostate cancer? | Q1 |  |
| What triggers are used for prostate biopsy in men with LUTS/BPE treated with 5ARIs? | Q4 |  |
| What concerns do urologists have regarding the impact that changing formulation has on PSA levels? | Q5a/b |  |

Q refers to the question number of the questionnaire and P refers to the question number of the PRF. A full list of questions is given in Supplementary Methods.

5ARI, 5-alpha reductase inhibitor; LUTS/BPE, lower urinary tract symptoms/benign prostate enlargement; PRF, patient record form; PSA, prostate-specific antigen. Each country was analyzed independently in this study.

SUPPLEMENTARY TABLE 2 Characteristics of urologists who completed the questionnaire.

|  | **Spain**  **(*N* = 100)** | **Brazil**  **(*N* = 100)** |
| --- | --- | --- |
| Mean years of experience (SD) | 16 (7.8) | 14 (8.8) |
| Median years of experience | 13 | 10 |
| Medical practice |  |  |
| Public | 87% | 35% |
| Private | 13% | 65% |
| Mean LUTS/BPE caseload (SD) |  |  |
| Number of patients managed or treated in the previous month | 149 (119.7) | 68 (87.6) |
| Number of patients currently treated with 5ARIs | 80 (79.8) | 54 (100.5) |

5ARI, 5-alpha reductase inhibitor; LUTS/BPE, lower urinary tract symptoms/benign prostate enlargement; SD, standard deviation. Each country was analyzed independently in this study.
